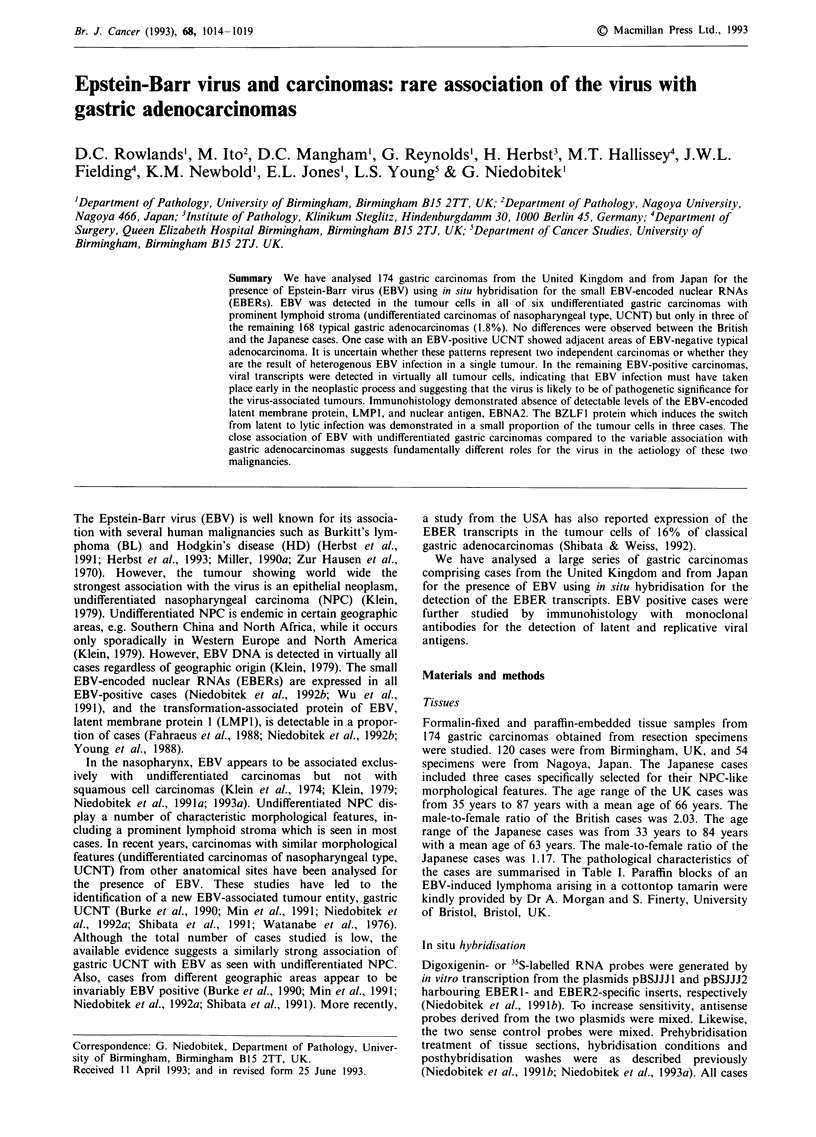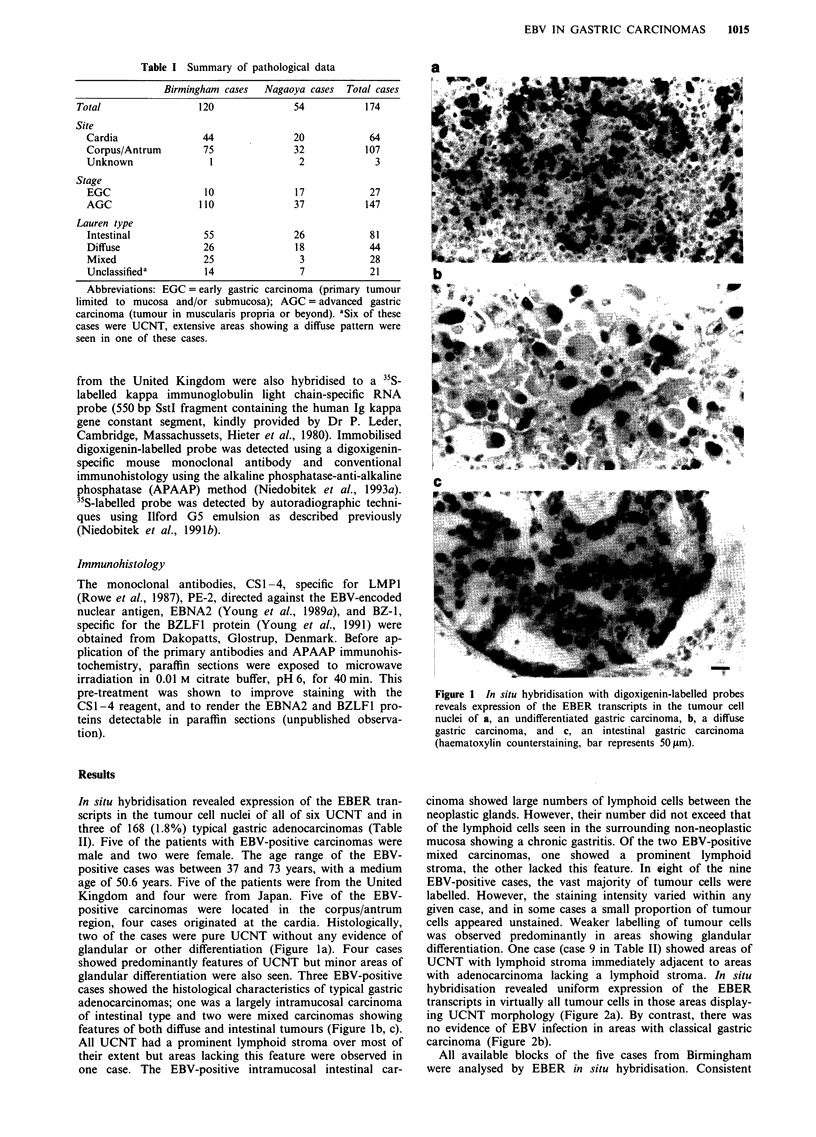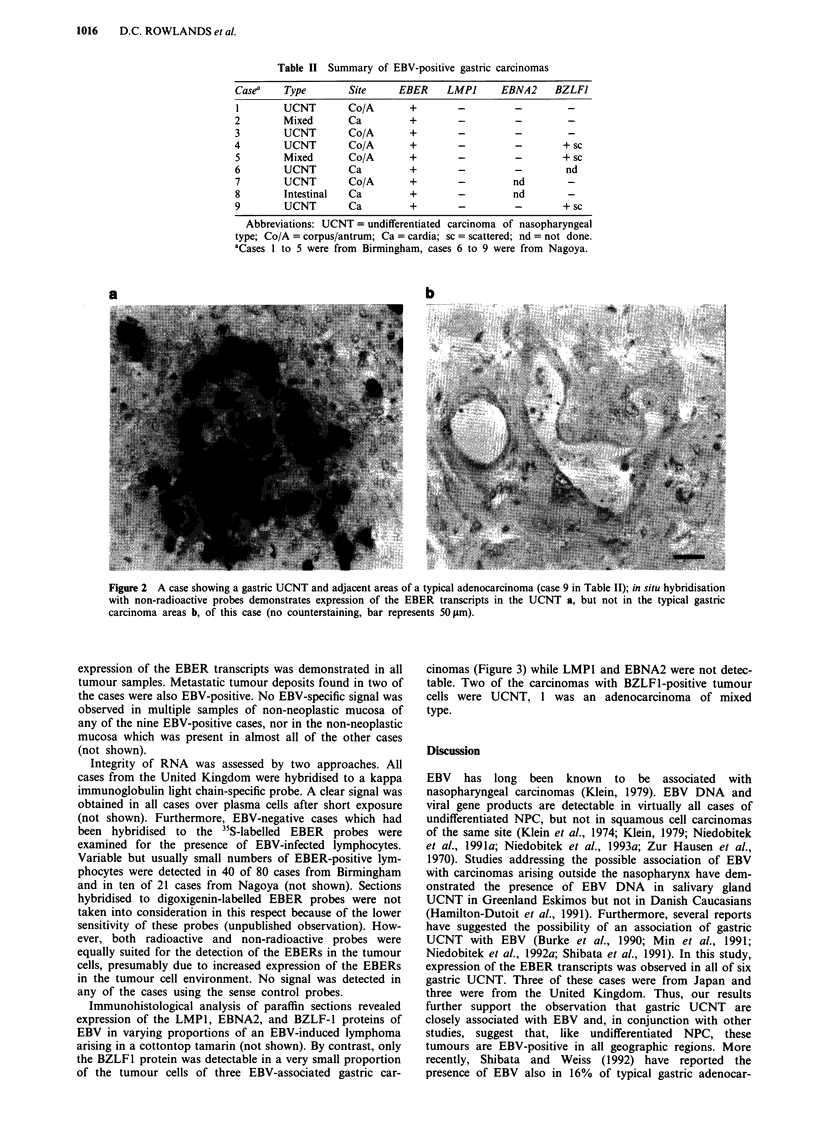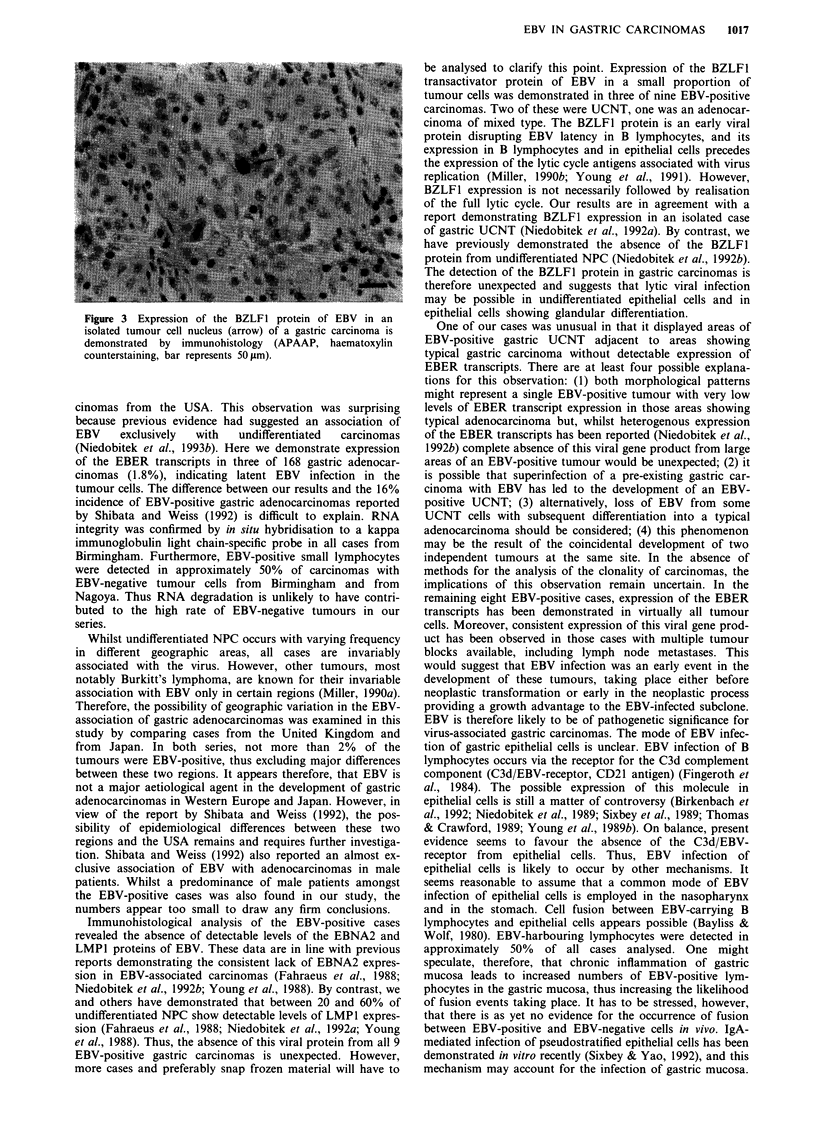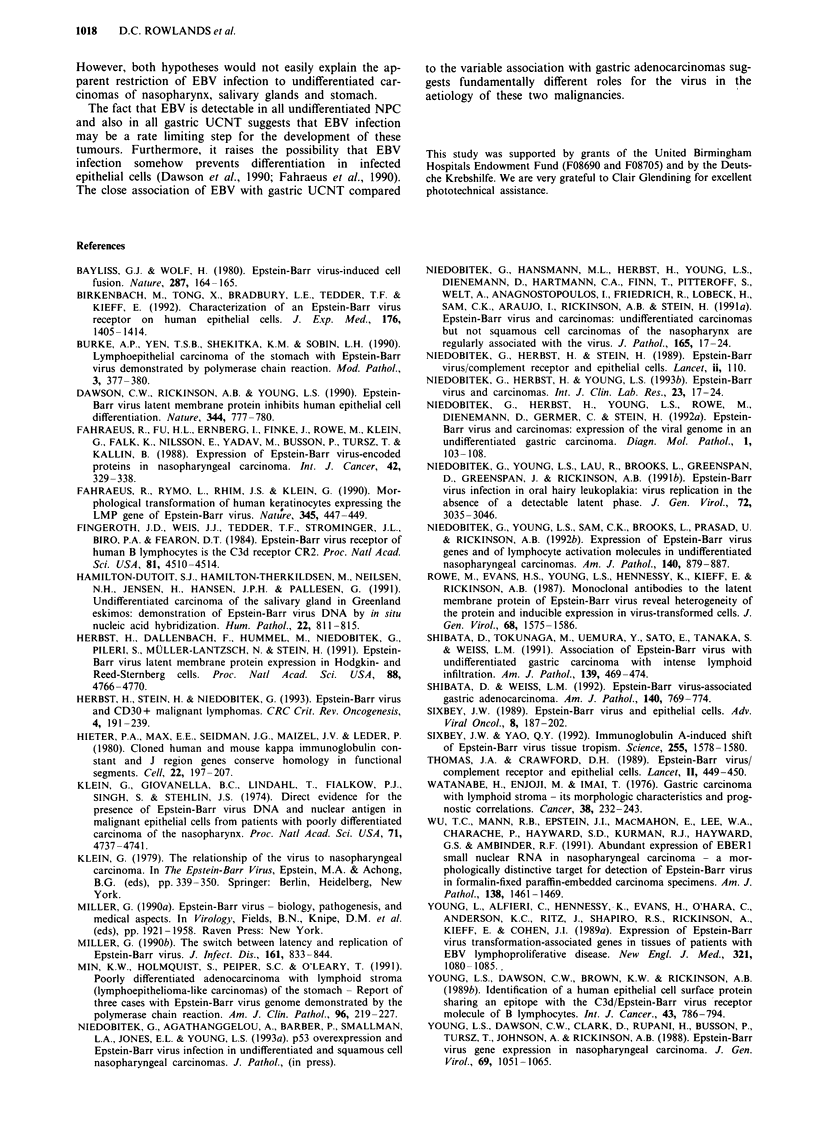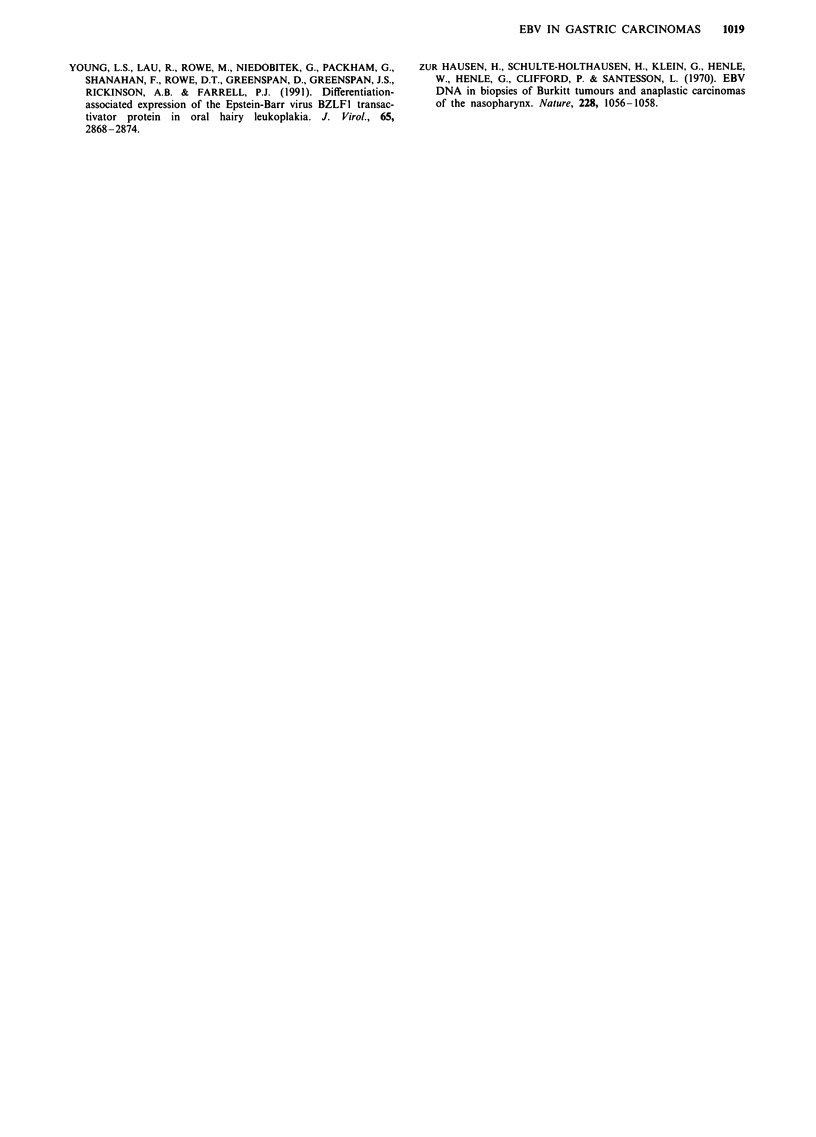# Epstein-Barr virus and carcinomas: rare association of the virus with gastric adenocarcinomas.

**DOI:** 10.1038/bjc.1993.472

**Published:** 1993-11

**Authors:** D. C. Rowlands, M. Ito, D. C. Mangham, G. Reynolds, H. Herbst, M. T. Hallissey, J. W. Fielding, K. M. Newbold, E. L. Jones, L. S. Young

**Affiliations:** Department of Pathology, University of Birmingham, UK.

## Abstract

**Images:**


Br. J. Cancer (1993), 68, 1014–1019 © Macmillan Press Ltd., 1993

### Epstein-Barr virus and carcinomas: rare association of the virus with gastric adenocarcinomas

D.C. Rowlands[1], M. Ito[2], D.C. Mangham[1], G. Reynolds[1], H. Herbst[3], M.T. Hallissey[4], J.W.L. Fielding[4], K.M. Newbold[1], E.L. Jones[1], L.S. Young[5] & G. Niedobitek[1]

[1]*Department of Pathology, University of Birmingham, Birmingham B15 2TT, UK;* [2]*Department of Pathology, Nagoya University, Nagoya 466, Japan;* [3]*Institute of Pathology, Klinikum Steglitz, Hindenburgdamm 30, 1000 Berlin 45, Germany;* [4]*Department of Surgery, Queen Elizabeth Hospital Birmingham, Birmingham B15 2TJ, UK;* [5]*Department of Cancer Studies, University of Birmingham, Birmingham B15 2TJ. UK.*

**Summary** We have analysed 174 gastric carcinomas from the United Kingdom and from Japan for the presence of Epstein-Barr virus (EBV) using *in situ* hybridisation for the small EBV-encoded nuclear RNAs (EBERs). EBV was detected in the tumour cells in all of six undifferentiated gastric carcinomas with prominent lymphoid stroma (undifferentiated carcinomas of nasopharyngeal type, UCNT) but only in three of the remaining 168 typical gastric adenocarcinomas (1.8%). No differences were observed between the British and the Japanese cases. One case with an EBV-positive UCNT showed adjacent areas of EBV-negative typical adenocarcinoma. It is uncertain whether these patterns represent two independent carcinomas or whether they are the result of heterogenous EBV infection in a single tumour. In the remaining EBV-positive carcinomas, viral transcripts were detected in virtually all tumour cells, indicating that EBV infection must have taken place early in the neoplastic process and suggesting that the virus is likely to be of pathogenetic significance for the virus-associated tumours. Immunohistology demonstrated absence of detectable levels of the EBV-encoded latent membrane protein, LMP1, and nuclear antigen, EBNA2. The BZLF1 protein which induces the switch from latent to lytic infection was demonstrated in a small proportion of the tumour cells in three cases. The close association of EBV with undifferentiated gastric carcinomas compared to the variable association with gastric adenocarcinomas suggests fundamentally different roles for the virus in the aetiology of these two malignancies.

The Epstein-Barr virus (EBV) is well known for its association with several human malignancies such as Burkitt's lymphoma (BL) and Hodgkin's disease (HD) (Herbst *et al.*, 1991; Herbst *et al.*, 1993; Miller, 1990*a*; Zur Hausen *et al.*, 1970). However, the tumour showing world wide the strongest association with the virus is an epithelial neoplasm, undifferentiated nasopharyngeal carcinoma (NPC) (Klein, 1979). Undifferentiated NPC is endemic in certain geographic areas, e.g. Southern China and North Africa, while it occurs only sporadically in Western Europe and North America (Klein, 1979). However, EBV DNA is detected in virtually all cases regardless of geographic origin (Klein, 1979). The small EBV-encoded nuclear RNAs (EBERs) are expressed in all EBV-positive cases (Niedobitek *et al.*, 1992*b*; Wu *et al.*, 1991), and the transformation-associated protein of EBV, latent membrane protein 1 (LMP1), is detectable in a proportion of cases (Fahraeus *et al.*, 1988; Niedobitek *et al.*, 1992*b*; Young *et al.*, 1988).

In the nasopharynx, EBV appears to be associated exclusively with undifferentiated carcinomas but not with squamous cell carcinomas (Klein *et al.*, 1974; Klein, 1979; Niedobitek *et al.*, 1991*a*; 1993*a*). Undifferentiated NPC display a number of characteristic morphological features, including a prominent lymphoid stroma which is seen in most cases. In recent years, carcinomas with similar morphological features (undifferentiated carcinomas of nasopharyngeal type, UCNT) from other anatomical sites have been analysed for the presence of EBV. These studies have led to the identification of a new EBV-associated tumour entity, gastric UCNT (Burke *et al.*, 1990; Min *et al.*, 1991; Niedobitek *et al.*, 1992*a*; Shibata *et al.*, 1991; Watanabe *et al.*, 1976). Although the total number of cases studied is low, the available evidence suggests a similarly strong association of gastric UCNT with EBV as seen with undifferentiated NPC. Also, cases from different geographic areas appear to be invariably EBV positive (Burke *et al.*, 1990; Min *et al.*, 1991; Niedobitek *et al.*, 1992*a*; Shibata *et al.*, 1991). More recently, a study from the USA has also reported expression of the EBER transcripts in the tumour cells of 16% of classical gastric adenocarcinomas (Shibata & Weiss, 1992).

We have analysed a large series of gastric carcinomas comprising cases from the United Kingdom and from Japan for the presence of EBV using *in situ* hybridisation for the detection of the EBER transcripts. EBV positive cases were further studied by immunohistology with monoclonal antibodies for the detection of latent and replicative viral antigens.

### Materials and methods

### Tissues

Formalin-fixed and paraffin-embedded tissue samples from 174 gastric carcinomas obtained from resection specimens were studied. 120 cases were from Birmingham, UK, and 54 specimens were from Nagoya, Japan. The Japanese cases included three cases specifically selected for their NPC-like morphological features. The age range of the UK cases was from 35 years to 87 years with a mean age of 66 years. The male-to-female ratio of the British cases was 2.03. The age range of the Japanese cases was from 33 years to 84 years with a mean age of 63 years. The male-to-female ratio of the Japanese cases was 1.17. The pathological characteristics of the cases are summarised in Table I. Paraffin blocks of an EBV-induced lymphoma arising in a cottontop tamarin were kindly provided by Dr A. Morgan and S. Finerty, University of Bristol, Bristol, UK.

### In situ *hybridisation*

Digoxigenin- or $^{35}$S-labelled RNA probes were generated by *in vitro* transcription from the plasmids pBSJJJ1 and pBSJJJ2 harbouring EBER1- and EBER2-specific inserts, respectively (Niedobitek *et al.*, 1991*b*). To increase sensitivity, antisense probes derived from the two plasmids were mixed. Likewise, the two sense control probes were mixed. Prehybridisation treatment of tissue sections, hybridisation conditions and posthybridisation washes were as described previously (Niedobitek *et al.*, 1991*b*; Niedobitek *et al.*, 1993*a*). All cases

Correspondence: G. Niedobitek, Department of Pathology, University of Birmingham, Birmingham B15 2TT, UK.
Received 11 April 1993; and in revised form 25 June 1993.

**Table I** Summary of pathological data

| | *Birmingham cases* | *Nagaoya cases* | *Total cases* |
|---|---|---|---|
| *Total* | 120 | 54 | 174 |
| *Site* | | | |
| Cardia | 44 | 20 | 64 |
| Corpus/Antrum | 75 | 32 | 107 |
| Unknown | 1 | 2 | 3 |
| *Stage* | | | |
| EGC | 10 | 17 | 27 |
| AGC | 110 | 37 | 147 |
| *Lauren type* | | | |
| Intestinal | 55 | 26 | 81 |
| Diffuse | 26 | 18 | 44 |
| Mixed | 25 | 3 | 28 |
| Unclassified[a] | 14 | 7 | 21 |

Abbreviations: EGC = early gastric carcinoma (primary tumour limited to mucosa and/or submucosa); AGC = advanced gastric carcinoma (tumour in muscularis propria or beyond). [a]Six of these cases were UCNT, extensive areas showing a diffuse pattern were seen in one of these cases.

from the United Kingdom were also hybridised to a $^{35}$S-labelled kappa immunoglobulin light chain-specific RNA probe (550 bp SstI fragment containing the human Ig kappa gene constant segment, kindly provided by Dr P. Leder, Cambridge, Massachussets, Hieter *et al.*, 1980). Immobilised digoxigenin-labelled probe was detected using a digoxigenin-specific mouse monoclonal antibody and conventional immunohistology using the alkaline phosphatase-anti-alkaline phosphatase (APAAP) method (Niedobitek *et al.*, 1993*a*). $^{35}$S-labelled probe was detected by autoradiographic techniques using Ilford G5 emulsion as described previously (Niedobitek *et al.*, 1991*b*).

### *Immunohistology*

The monoclonal antibodies, CS1-4, specific for LMP1 (Rowe *et al.*, 1987), PE-2, directed against the EBV-encoded nuclear antigen, EBNA2 (Young *et al.*, 1989*a*), and BZ-1, specific for the BZLF1 protein (Young *et al.*, 1991) were obtained from Dakopatts, Glostrup, Denmark. Before application of the primary antibodies and APAAP immunohistochemistry, paraffin sections were exposed to microwave irradiation in 0.01 M citrate buffer, pH 6, for 40 min. This pre-treatment was shown to improve staining with the CS1-4 reagent, and to render the EBNA2 and BZLF1 proteins detectable in paraffin sections (unpublished observation).

**Figure 1** *In situ* hybridisation with digoxigenin-labelled probes reveals expression of the EBER transcripts in the tumour cell nuclei of **a**, an undifferentiated gastric carcinoma, **b**, a diffuse gastric carcinoma, and **c**, an intestinal gastric carcinoma (haematoxylin counterstaining, bar represents 50 μm).

### Results

*In situ* hybridisation revealed expression of the EBER transcripts in the tumour cell nuclei of all of six UCNT and in three of 168 (1.8%) typical gastric adenocarcinomas (Table II). Five of the patients with EBV-positive carcinomas were male and two were female. The age range of the EBV-positive cases was between 37 and 73 years, with a medium age of 50.6 years. Five of the patients were from the United Kingdom and four were from Japan. Five of the EBV-positive carcinomas were located in the corpus/antrum region, four cases originated at the cardia. Histologically, two of the cases were pure UCNT without any evidence of glandular or other differentiation (Figure 1a). Four cases showed predominantly features of UCNT but minor areas of glandular differentiation were also seen. Three EBV-positive cases showed the histological characteristics of typical gastric adenocarcinomas; one was a largely intramucosal carcinoma of intestinal type and two were mixed carcinomas showing features of both diffuse and intestinal tumours (Figure 1b, c). All UCNT had a prominent lymphoid stroma over most of their extent but areas lacking this feature were observed in one case. The EBV-positive intramucosal intestinal carcinoma showed large numbers of lymphoid cells between the neoplastic glands. However, their number did not exceed that of the lymphoid cells seen in the surrounding non-neoplastic mucosa showing a chronic gastritis. Of the two EBV-positive mixed carcinomas, one showed a prominent lymphoid stroma, the other lacked this feature. In eight of the nine EBV-positive cases, the vast majority of tumour cells were labelled. However, the staining intensity varied within any given case, and in some cases a small proportion of tumour cells appeared unstained. Weaker labelling of tumour cells was observed predominantly in areas showing glandular differentiation. One case (case 9 in Table II) showed areas of UCNT with lymphoid stroma immediately adjacent to areas with adenocarcinoma lacking a lymphoid stroma. *In situ* hybridisation revealed uniform expression of the EBER transcripts in virtually all tumour cells in those areas displaying UCNT morphology (Figure 2a). By contrast, there was no evidence of EBV infection in areas with classical gastric carcinoma (Figure 2b).

All available blocks of the five cases from Birmingham were analysed by EBER *in situ* hybridisation. Consistent

**Table II** Summary of EBV-positive gastric carcinomas

| *Case*[a] | *Type* | *Site* | *EBER* | *LMP1* | *EBNA2* | *BZLF1* |
|---|---|---|---|---|---|---|
| 1 | UCNT | Co/A | + | – | – | – |
| 2 | Mixed | Ca | + | – | – | – |
| 3 | UCNT | Co/A | + | – | – | – |
| 4 | UCNT | Co/A | + | – | – | + sc |
| 5 | Mixed | Co/A | + | – | – | + sc |
| 6 | UCNT | Ca | + | – | – | nd |
| 7 | UCNT | Co/A | + | – | nd | – |
| 8 | Intestinal | Ca | + | – | nd | – |
| 9 | UCNT | Ca | + | – | – | + sc |

Abbreviations: UCNT = undifferentiated carcinoma of nasopharyngeal type; Co/A = corpus/antrum; Ca = cardia; sc = scattered; nd = not done. [a]Cases 1 to 5 were from Birmingham, cases 6 to 9 were from Nagoya.

**Figure 2** A case showing a gastric UCNT and adjacent areas of a typical adenocarcinoma (case 9 in Table II); *in situ* hybridisation with non-radioactive probes demonstrates expression of the EBER transcripts in the UCNT **a**, but not in the typical gastric carcinoma areas **b**, of this case (no counterstaining, bar represents 50 μm).

expression of the EBER transcripts was demonstrated in all tumour samples. Metastatic tumour deposits found in two of the cases were also EBV-positive. No EBV-specific signal was observed in multiple samples of non-neoplastic mucosa of any of the nine EBV-positive cases, nor in the non-neoplastic mucosa which was present in almost all of the other cases (not shown).

Integrity of RNA was assessed by two approaches. All cases from the United Kingdom were hybridised to a kappa immunoglobulin light chain-specific probe. A clear signal was obtained in all cases over plasma cells after short exposure (not shown). Furthermore, EBV-negative cases which had been hybridised to the $^{35}$S-labelled EBER probes were examined for the presence of EBV-infected lymphocytes. Variable but usually small numbers of EBER-positive lymphocytes were detected in 40 of 80 cases from Birmingham and in ten of 21 cases from Nagoya (not shown). Sections hybridised to digoxigenin-labelled EBER probes were not taken into consideration in this respect because of the lower sensitivity of these probes (unpublished observation). However, both radioactive and non-radioactive probes were equally suited for the detection of the EBERs in the tumour cells, presumably due to increased expression of the EBERs in the tumour cell environment. No signal was detected in any of the cases using the sense control probes.

Immunohistological analysis of paraffin sections revealed expression of the LMP1, EBNA2, and BZLF-1 proteins of EBV in varying proportions of an EBV-induced lymphoma arising in a cottontop tamarin (not shown). By contrast, only the BZLF1 protein was detectable in a very small proportion of the tumour cells of three EBV-associated gastric carcinomas (Figure 3) while LMP1 and EBNA2 were not detectable. Two of the carcinomas with BZLF1-positive tumour cells were UCNT, 1 was an adenocarcinoma of mixed type.

### Discussion

EBV has long been known to be associated with nasopharyngeal carcinomas (Klein, 1979). EBV DNA and viral gene products are detectable in virtually all cases of undifferentiated NPC, but not in squamous cell carcinomas of the same site (Klein *et al*., 1974; Klein, 1979; Niedobitek *et al*., 1991*a*; Niedobitek *et al*., 1993*a*; Zur Hausen *et al*., 1970). Studies addressing the possible association of EBV with carcinomas arising outside the nasopharynx have demonstrated the presence of EBV DNA in salivary gland UCNT in Greenland Eskimos but not in Danish Caucasians (Hamilton-Dutoit *et al*., 1991). Furthermore, several reports have suggested the possibility of an association of gastric UCNT with EBV (Burke *et al*., 1990; Min *et al*., 1991; Niedobitek *et al*., 1992*a*; Shibata *et al*., 1991). In this study, expression of the EBER transcripts was observed in all of six gastric UCNT. Three of these cases were from Japan and three were from the United Kingdom. Thus, our results further support the observation that gastric UCNT are closely associated with EBV and, in conjunction with other studies, suggest that, like undifferentiated NPC, these tumours are EBV-positive in all geographic regions. More recently, Shibata and Weiss (1992) have reported the presence of EBV also in 16% of typical gastric adenocar-

**Figure 3** Expression of the BZLF1 protein of EBV in an isolated tumour cell nucleus (arrow) of a gastric carcinoma is demonstrated by immunohistology (APAAP, haematoxylin counterstaining, bar represents 50 μm).

cinomas from the USA. This observation was surprising because previous evidence had suggested an association of EBV exclusively with undifferentiated carcinomas (Niedobitek *et al.*, 1993*b*). Here we demonstrate expression of the EBER transcripts in three of 168 gastric adenocarcinomas (1.8%), indicating latent EBV infection in the tumour cells. The difference between our results and the 16% incidence of EBV-positive gastric adenocarcinomas reported by Shibata and Weiss (1992) is difficult to explain. RNA integrity was confirmed by *in situ* hybridisation to a kappa immunoglobulin light chain-specific probe in all cases from Birmingham. Furthermore, EBV-positive small lymphocytes were detected in approximately 50% of carcinomas with EBV-negative tumour cells from Birmingham and from Nagoya. Thus RNA degradation is unlikely to have contributed to the high rate of EBV-negative tumours in our series.

Whilst undifferentiated NPC occurs with varying frequency in different geographic areas, all cases are invariably associated with the virus. However, other tumours, most notably Burkitt's lymphoma, are known for their invariable association with EBV only in certain regions (Miller, 1990*a*). Therefore, the possibility of geographic variation in the EBV-association of gastric adenocarcinomas was examined in this study by comparing cases from the United Kingdom and from Japan. In both series, not more than 2% of the tumours were EBV-positive, thus excluding major differences between these two regions. It appears therefore, that EBV is not a major aetiological agent in the development of gastric adenocarcinomas in Western Europe and Japan. However, in view of the report by Shibata and Weiss (1992), the possibility of epidemiological differences between these two regions and the USA remains and requires further investigation. Shibata and Weiss (1992) also reported an almost exclusive association of EBV with adenocarcinomas in male patients. Whilst a predominance of male patients amongst the EBV-positive cases was also found in our study, the numbers appear too small to draw any firm conclusions.

Immunohistological analysis of the EBV-positive cases revealed the absence of detectable levels of the EBNA2 and LMP1 proteins of EBV. These data are in line with previous reports demonstrating the consistent lack of EBNA2 expression in EBV-associated carcinomas (Fahraeus *et al.*, 1988; Niedobitek *et al.*, 1992*b*; Young *et al.*, 1988). By contrast, we and others have demonstrated that between 20 and 60% of undifferentiated NPC show detectable levels of LMP1 expression (Fahraeus *et al.*, 1988; Niedobitek *et al.*, 1992*a*; Young *et al.*, 1988). Thus, the absence of this viral protein from all 9 EBV-positive gastric carcinomas is unexpected. However, more cases and preferably snap frozen material will have to be analysed to clarify this point. Expression of the BZLF1 transactivator protein of EBV in a small proportion of tumour cells was demonstrated in three of nine EBV-positive carcinomas. Two of these were UCNT, one was an adenocarcinoma of mixed type. The BZLF1 protein is an early viral protein disrupting EBV latency in B lymphocytes, and its expression in B lymphocytes and in epithelial cells precedes the expression of the lytic cycle antigens associated with virus replication (Miller, 1990*b*; Young *et al.*, 1991). However, BZLF1 expression is not necessarily followed by realisation of the full lytic cycle. Our results are in agreement with a report demonstrating BZLF1 expression in an isolated case of gastric UCNT (Niedobitek *et al.*, 1992*a*). By contrast, we have previously demonstrated the absence of the BZLF1 protein from undifferentiated NPC (Niedobitek *et al.*, 1992*b*). The detection of the BZLF1 protein in gastric carcinomas is therefore unexpected and suggests that lytic viral infection may be possible in undifferentiated epithelial cells and in epithelial cells showing glandular differentiation.

One of our cases was unusual in that it displayed areas of EBV-positive gastric UCNT adjacent to areas showing typical gastric carcinoma without detectable expression of EBER transcripts. There are at least four possible explanations for this observation: (1) both morphological patterns might represent a single EBV-positive tumour with very low levels of EBER transcript expression in those areas showing typical adenocarcinoma but, whilst heterogenous expression of the EBER transcripts has been reported (Niedobitek *et al.*, 1992*b*) complete absence of this viral gene product from large areas of an EBV-positive tumour would be unexpected; (2) it is possible that superinfection of a pre-existing gastric carcinoma with EBV has led to the development of an EBV-positive UCNT; (3) alternatively, loss of EBV from some UCNT cells with subsequent differentiation into a typical adenocarcinoma should be considered; (4) this phenomenon may be the result of the coincidental development of two independent tumours at the same site. In the absence of methods for the analysis of the clonality of carcinomas, the implications of this observation remain uncertain. In the remaining eight EBV-positive cases, expression of the EBER transcripts has been demonstrated in virtually all tumour cells. Moreover, consistent expression of this viral gene product has been observed in those cases with multiple tumour blocks available, including lymph node metastases. This would suggest that EBV infection was an early event in the development of these tumours, taking place either before neoplastic transformation or early in the neoplastic process providing a growth advantage to the EBV-infected subclone. EBV is therefore likely to be of pathogenetic significance for virus-associated gastric carcinomas. The mode of EBV infection of gastric epithelial cells is unclear. EBV infection of B lymphocytes occurs via the receptor for the C3d complement component (C3d/EBV-receptor, CD21 antigen) (Fingeroth *et al.*, 1984). The possible expression of this molecule in epithelial cells is still a matter of controversy (Birkenbach *et al.*, 1992; Niedobitek *et al.*, 1989; Sixbey *et al.*, 1989; Thomas & Crawford, 1989; Young *et al.*, 1989*b*). On balance, present evidence seems to favour the absence of the C3d/EBV-receptor from epithelial cells. Thus, EBV infection of epithelial cells is likely to occur by other mechanisms. It seems reasonable to assume that a common mode of EBV infection of epithelial cells is employed in the nasopharynx and in the stomach. Cell fusion between EBV-carrying B lymphocytes and epithelial cells appears possible (Bayliss & Wolf, 1980). EBV-harbouring lymphocytes were detected in approximately 50% of all cases analysed. One might speculate, therefore, that chronic inflammation of gastric mucosa leads to increased numbers of EBV-positive lymphocytes in the gastric mucosa, thus increasing the likelihood of fusion events taking place. It has to be stressed, however, that there is as yet no evidence for the occurrence of fusion between EBV-positive and EBV-negative cells *in vivo*. IgA-mediated infection of pseudostratified epithelial cells has been demonstrated *in vitro* recently (Sixbey & Yao, 1992), and this mechanism may account for the infection of gastric mucosa.

However, both hypotheses would not easily explain the apparent restriction of EBV infection to undifferentiated carcinomas of nasopharynx, salivary glands and stomach.

The fact that EBV is detectable in all undifferentiated NPC and also in all gastric UCNT suggests that EBV infection may be a rate limiting step for the development of these tumours. Furthermore, it raises the possibility that EBV infection somehow prevents differentiation in infected epithelial cells (Dawson *et al.*, 1990; Fahraeus *et al.*, 1990). The close association of EBV with gastric UCNT compared to the variable association with gastric adenocarcinomas suggests fundamentally different roles for the virus in the aetiology of these two malignancies.

This study was supported by grants of the United Birmingham Hospitals Endowment Fund (F08690 and F08705) and by the Deutsche Krebshilfe. We are very grateful to Clair Glendining for excellent phototechnical assistance.

### References

BAYLISS, G.J. & WOLF, H. (1980). Epstein-Barr virus-induced cell fusion. *Nature*, **287**, 164-165.

BIRKENBACH, M., TONG, X., BRADBURY, L.E., TEDDER, T.F. & KIEFF, E. (1992). Characterization of an Epstein-Barr virus receptor on human epithelial cells. *J. Exp. Med.*, **176**, 1405-1414.

BURKE, A.P., YEN, T.S.B., SHEKITKA, K.M. & SOBIN, L.H. (1990). Lymphoepithelial carcinoma of the stomach with Epstein-Barr virus demonstrated by polymerase chain reaction. *Mod. Pathol.*, **3**, 377-380.

DAWSON, C.W., RICKINSON, A.B. & YOUNG, L.S. (1990). Epstein-Barr virus latent membrane protein inhibits human epithelial cell differentiation. *Nature*, **344**, 777-780.

FAHRAEUS, R., FU, H.L., ERNBERG, I., FINKE, J., ROWE, M., KLEIN, G., FALK, K., NILSSON, E., YADAV, M., BUSSON, P., TURSZ, T. & KALLIN, B. (1988). Expression of Epstein-Barr virus-encoded proteins in nasopharyngeal carcinoma. *Int. J. Cancer*, **42**, 329-338.

FAHRAEUS, R., RYMO, L., RHIM, J.S. & KLEIN, G. (1990). Morphological transformation of human keratinocytes expressing the LMP gene of Epstein-Barr virus. *Nature*, **345**, 447-449.

FINGEROTH, J.D., WEIS, J.J., TEDDER, T.F., STROMINGER, J.L., BIRO, P.A. & FEARON, D.T. (1984). Epstein-Barr virus receptor of human B lymphocytes is the C3d receptor CR2. *Proc. Natl Acad. Sci. USA*, **81**, 4510-4514.

HAMILTON-DUTOIT, S.J., HAMILTON-THERKILDSEN, M., NEILSEN, N.H., JENSEN, H., HANSEN, J.P.H. & PALLESEN, G. (1991). Undifferentiated carcinoma of the salivary gland in Greenland eskimos: demonstration of Epstein-Barr virus DNA by *in situ* nucleic acid hybridization. *Hum. Pathol.*, **22**, 811-815.

HERBST, H., DALLENBACH, F., HUMMEL, M., NIEDOBITEK, G., PILERI, S., MÜLLER-LANTZSCH, N. & STEIN, H. (1991). Epstein-Barr virus latent membrane protein expression in Hodgkin- and Reed-Sternberg cells. *Proc. Natl Acad. Sci. USA*, **88**, 4766-4770.

HERBST, H., STEIN, H. & NIEDOBITEK, G. (1993). Epstein-Barr virus and CD30+ malignant lymphomas. *CRC Crit. Rev. Oncogenesis*, **4**, 191-239.

HIETER, P.A., MAX, E.E., SEIDMAN, J.G., MAIZEL, J.V. & LEDER, P. (1980). Cloned human and mouse kappa immunoglobulin constant and J region genes conserve homology in functional segments. *Cell*, **22**, 197-207.

KLEIN, G., GIOVANELLA, B.C., LINDAHL, T., FIALKOW, P.J., SINGH, S. & STEHLIN, J.S. (1974). Direct evidence for the presence of Epstein-Barr virus DNA and nuclear antigen in malignant epithelial cells from patients with poorly differentiated carcinoma of the nasopharynx. *Proc. Natl Acad. Sci. USA*, **71**, 4737-4741.

KLEIN, G. (1979). The relationship of the virus to nasopharyngeal carcinoma. In *The Epstein-Barr Virus*, Epstein, M.A. & Achong, B.G. (eds), pp. 339-350. Springer: Berlin, Heidelberg, New York.

MILLER, G. (1990*a*). Epstein-Barr virus - biology, pathogenesis, and medical aspects. In *Virology*, Fields, B.N., Knipe, D.M. *et al.* (eds), pp. 1921-1958. Raven Press: New York.

MILLER, G. (1990*b*). The switch between latency and replication of Epstein-Barr virus. *J. Infect. Dis.*, **161**, 833-844.

MIN, K.W., HOLMQUIST, S., PEIPER, S.C. & O'LEARY, T. (1991). Poorly differentiated adenocarcinoma with lymphoid stroma (lymphoepithelioma-like carcinomas) of the stomach - Report of three cases with Epstein-Barr virus genome demonstrated by the polymerase chain reaction. *Am. J. Clin. Pathol.*, **96**, 219-227.

NIEDOBITEK, G., AGATHANGGELOU, A., BARBER, P., SMALLMAN, L.A., JONES, E.L. & YOUNG, L.S. (1993*a*). p53 overexpression and Epstein-Barr virus infection in undifferentiated and squamous cell nasopharyngeal carcinomas. *J. Pathol.*, (in press).

NIEDOBITEK, G., HANSMANN, M.L., HERBST, H., YOUNG, L.S., DIENEMANN, D., HARTMANN, C.A., FINN, T., PITTEROFF, S., WELT, A., ANAGNOSTOPOULOS, I., FRIEDRICH, R., LOBECK, H., SAM, C.K., ARAUJO, I., RICKINSON, A.B. & STEIN, H. (1991*a*). Epstein-Barr virus and carcinomas: undifferentiated carcinomas but not squamous cell carcinomas of the nasopharynx are regularly associated with the virus. *J. Pathol.*, **165**, 17-24.

NIEDOBITEK, G., HERBST, H. & STEIN, H. (1989). Epstein-Barr virus/complement receptor and epithelial cells. *Lancet*, **ii**, 110.

NIEDOBITEK, G., HERBST, H. & YOUNG, L.S. (1993*b*). Epstein-Barr virus and carcinomas. *Int. J. Clin. Lab. Res.*, **23**, 17-24.

NIEDOBITEK, G., HERBST, H., YOUNG, L.S., ROWE, M., DIENEMANN, D., GERMER, C. & STEIN, H. (1992*a*). Epstein-Barr virus and carcinomas: expression of the viral genome in an undifferentiated gastric carcinoma. *Diagn. Mol. Pathol.*, **1**, 103-108.

NIEDOBITEK, G., YOUNG, L.S., LAU, R., BROOKS, L., GREENSPAN, D., GREENSPAN, J. & RICKINSON, A.B. (1991*b*). Epstein-Barr virus infection in oral hairy leukoplakia: virus replication in the absence of a detectable latent phase. *J. Gen. Virol.*, **72**, 3035-3046.

NIEDOBITEK, G., YOUNG, L.S., SAM, C.K., BROOKS, L., PRASAD, U. & RICKINSON, A.B. (1992*b*). Expression of Epstein-Barr virus genes and of lymphocyte activation molecules in undifferentiated nasopharyngeal carcinomas. *Am. J. Pathol.*, **140**, 879-887.

ROWE, M., EVANS, H.S., YOUNG, L.S., HENNESSY, K., KIEFF, E. & RICKINSON, A.B. (1987). Monoclonal antibodies to the latent membrane protein of Epstein-Barr virus reveal heterogeneity of the protein and inducible expression in virus-transformed cells. *J. Gen. Virol.*, **68**, 1575-1586.

SHIBATA, D., TOKUNAGA, M., UEMURA, Y., SATO, E., TANAKA, S. & WEISS, L.M. (1991). Association of Epstein-Barr virus with undifferentiated gastric carcinoma with intense lymphoid infiltration. *Am. J. Pathol.*, **139**, 469-474.

SHIBATA, D. & WEISS, L.M. (1992). Epstein-Barr virus-associated gastric adenocarcinoma. *Am. J. Pathol.*, **140**, 769-774.

SIXBEY, J.W. (1989). Epstein-Barr virus and epithelial cells. *Adv. Viral Oncol.*, **8**, 187-202.

SIXBEY, J.W. & YAO, Q.Y. (1992). Immunoglobulin A-induced shift of Epstein-Barr virus tissue tropism. *Science*, **255**, 1578-1580.

THOMAS, J.A. & CRAWFORD, D.H. (1989). Epstein-Barr virus/complement receptor and epithelial cells. *Lancet*, **II**, 449-450.

WATANABE, H., ENJOJI, M. & IMAI, T. (1976). Gastric carcinoma with lymphoid stroma - its morphologic characteristics and prognostic correlations. *Cancer*, **38**, 232-243.

WU, T.C., MANN, R.B., EPSTEIN, J.I., MACMAHON, E., LEE, W.A., CHARACHE, P., HAYWARD, S.D., KURMAN, R.J., HAYWARD, G.S. & AMBINDER, R.F. (1991). Abundant expression of EBER1 small nuclear RNA in nasopharyngeal carcinoma - a morphologically distinctive target for detection of Epstein-Barr virus in formalin-fixed paraffin-embedded carcinoma specimens. *Am. J. Pathol.*, **138**, 1461-1469.

YOUNG, L., ALFIERI, C., HENNESSY, K., EVANS, H., O'HARA, C., ANDERSON, K.C., RITZ, J., SHAPIRO, R.S., RICKINSON, A., KIEFF, E. & COHEN, J.I. (1989*a*). Expression of Epstein-Barr virus transformation-associated genes in tissues of patients with EBV lymphoproliferative disease. *New Engl. J. Med.*, **321**, 1080-1085.

YOUNG, L.S., DAWSON, C.W., BROWN, K.W. & RICKINSON, A.B. (1989*b*). Identification of a human epithelial cell surface protein sharing an epitope with the C3d/Epstein-Barr virus receptor molecule of B lymphocytes. *Int. J. Cancer.*, **43**, 786-794.

YOUNG, L.S., DAWSON, C.W., CLARK, D., RUPANI, H., BUSSON, P., TURSZ, T., JOHNSON, A. & RICKINSON, A.B. (1988). Epstein-Barr virus gene expression in nasopharyngeal carcinoma. *J. Gen. Virol.*, **69**, 1051-1065.

YOUNG, L.S., LAU, R., ROWE, M., NIEDOBITEK, G., PACKHAM, G., SHANAHAN, F., ROWE, D.T., GREENSPAN, D., GREENSPAN, J.S., RICKINSON, A.B. & FARRELL, P.J. (1991). Differentiation-associated expression of the Epstein-Barr virus BZLF1 transactivator protein in oral hairy leukoplakia. *J. Virol.*, **65**, 2868–2874.

ZUR HAUSEN, H., SCHULTE-HOLTHAUSEN, H., KLEIN, G., HENLE, W., HENLE, G., CLIFFORD, P. & SANTESSON, L. (1970). EBV DNA in biopsies of Burkitt tumours and anaplastic carcinomas of the nasopharynx. *Nature*, **228**, 1056–1058.