# Epstein-Barr virus and carcinomas: rare association of the virus with gastric adenocarcinomas

**DOI:** 10.1038/bjc.2016.156

**Published:** 2016-05-26

**Authors:** D C Rowlands, M Ito, D C Mangham, G Reynolds, H Herbst, M T Hallissey, J W L Fielding, K M Newbold, E L Jones, L S Young, G Niedobitek

**Correction to:** *British Journal of Cancer* (1993) **68**, 1014–1019; doi:10.1038/bjc.1993.472; published online November 1993

The corresponding author of the above paper, G Niedobitek, has informed us that, despite his name appearing correctly in the original printed paper and hence in the online PDF version, his name was omitted from the list of authors accompanying the paper’s online Abstract and in the online Table of Contents for this issue. The publishers would like to apologise for this mistake. G Niedobitek appears correctly in the author list above.

